# Association between oxidative balance score and gallstone disease: a population-based study from NHANES

**DOI:** 10.3389/fnut.2025.1539969

**Published:** 2025-01-22

**Authors:** Mengmeng Zhang, Aiming Yang

**Affiliations:** Department of Gastroenterology, Peking Union Medical College Hospital, Chinese Academy of Medical Sciences and Peking Union Medical College, Beijing, China

**Keywords:** oxidative balance score, diet, lifestyle, gallstones, NHANES

## Abstract

**Background:**

Oxidative stress has been reported to participant in the pathogenesis of gallstones. Oxidative balance score (OBS) represents pro-oxidant and antioxidant exposures to diet and lifestyle, closely associated with multiple metabolic disorders. However, the relationship between OBS and gallstones remains unclear.

**Methods:**

This study analyzed cross-sectional data from the National Health and Nutrition Examination Survey (NHANES) 2017–2020. OBS was calculated based on the 24-h recall interviews or questionnaires. We used weighted logistic regression, restricted cubic splines (RCS), weighted quantile sum (WQS) regression and the Bayesian kernel machine regression (BKMR) model to identify the relationship between OBS and gallstones. Subgroup analysis and sensitivity analysis were used to explore potential heterogeneity and stability of the results. Mediation analysis was performed to assess the mediating effects of serum lipid in the association between OBS and gallstones.

**Results:**

A total of 7,618 participants were finally included in this study. Weighted logistics regression showed that total OBS was associated with gallstones risk (OR = 0.98, *p* = 0.03), particularly in individuals who were under 60 years old, Hispanic, educated below high school, non-smokers, had hypertension or malignancy. Dietary and lifestyle OBS independently contribute to the protection against gallstones. RCS analysis indicated a non-linear relationship between OBS and gallstones (*p* = 0.03). WQS and BKMR model identified that BMI, vitamin E, vitamin B6, magnesium and carotene played relatively important role among 20 components. Mediation analysis showed serum TG and HDL as mediators of the association between OBS and gallstones.

**Conclusion:**

Higher OBS or increased oxidative balance are positively associated with reduction of gallstone risk. This findings provide valuable insights for surveillance and interventions targeting for antioxidant-rich diet and lifestyle for gallstone disease.

## Introduction

Cholelithiasis or gallstone disease is a common digestive disease and causes significant health care burden worldwide. The prevalence of gallstones is approximately 10–15% in adult population, which is usually underestimated as about 70% of patients with cholelithiasis are asymptomatic (1). About 10–20% of patients have varying degrees of abdominal symptoms or even develop severe complications (2), such as gallstone ileus especially in the elderly (3). Gallstones are traditionally classified as cholesterol, pigment, or mixed stones based on their composition. The mechanism of gallstone formation has not been well understood, involving multiple factors. Disrupted cholesterol homeostasis is the primary cause of cholesterol gallstone formation and depends largely on genetic predisposition, while pigment gallstones are the integrated consequences of hepatic hypersection of bilirubin, bile stasis, and bacterial infection (4). Besides, age, female, ethnic background, dietary or lifestyle habits, bariatric surgery-induced rapid weight loss are the modifiable risk factors, particularly in the development of cholesterol gallstones (1, 5, 6).

Oxidative stress or imbalance plays an important role in multiple physiological processes and has been identified to participant in the nucleation and deposition of gallstones (7). Abnormal oxidative stress may induce dysfunction of lipid metabolism through affecting metabolism-related organelles and leading to cellular damage, lipid peroxidation, and mitochondrial dysfunction, etc (8). In addition, Sanikidze et al. (7) revealed the existence of oxidized bilirubin free radical in pigmented gallstones using electron paramagnetic resonance, acting as a key driver for gallstone formation. Besides maintained mainly by endogenous enzymatic mechanisms, the pro-oxidant/antioxidant balance is also affected by exogenous factors, including diet and lifestyle (9). Oxidative balance score (OBS) is a novel concept to assess pro-oxidant and antioxidant exposures in individuals, which was composed of dietary intakes and lifestyles (9, 10). Higher OBS represents a stronger antioxidant capacity within the body. Several studies reported that OBS is significantly associated with metabolic disorders (11), cardiovascular diseases (12), chronic disease (13, 14), or malignant disease (15). However, the potential association between OBS and gallstones remains unclear. The joint and independent effects of OBS components on gallstones should be evaluated to comprehensively reveal the role of pro-oxidant and antioxidant balance adjusted by diary diet or lifestyle in the pathogenesis of gallstones, and provide evidence for gallstone prevention through maintaining antioxidant-rich diet or lifestyle.

Therefore, we aimed to investigate the association between dietary and lifestyle integrated OBS and all types of gallstones incidence in the general UC population from the National Health and Nutrition Examination Survey (NHANES) 2017 to March 2020 cycle to provide further evidence for the occurrence of gallstones.

## Materials and methods

### Study design and participants

**Figure 1:**
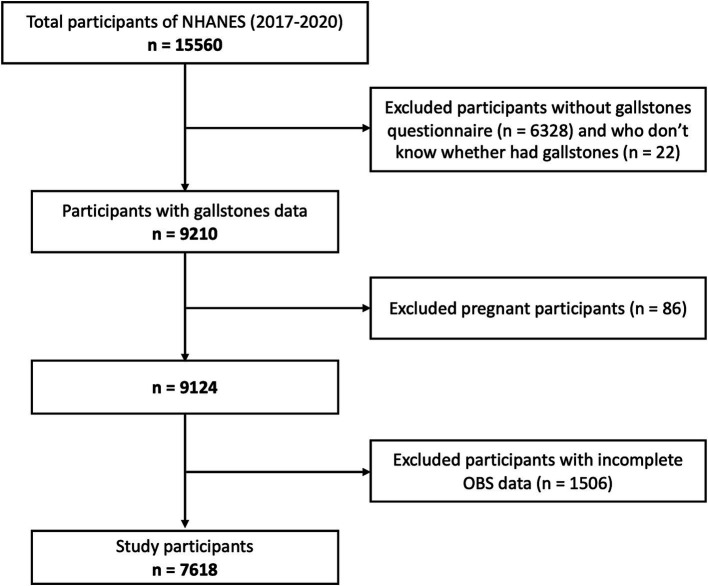
Study flowchart of participants. According to the inclusion and exclusion criteria, 7,618 of 15,560 NAHNES participants between 2017 and 2020 were finally included. NHANES, National Health and Nutrition Examination Survey; OBS, Oxidative balance score.

All data were obtained from NHANES 2017-March 2020, a nationally population-based survey conducted by the National Center for Health Statistics (NCHS) of the centers to evaluate the nutritional status and health of adults and children in the US. As a large-scale, national study, the rigorous stratified, multistage probability sampling method ensures the comprehensive representativeness and diversity of the participants, which has superior performance in the assessment of risk factors. Initially, 9,232 of 15,560 individuals completed the questionnaire of whether they had gallstones. As is shown in Figure 1, 86 pregnant participants, 22 participants with incomplete gallstone questionnaires, 1,506 participants with incomplete data of OBS were excluded. Finally, 7,618 participants were included in this study, who were all ≥20 years old. The National Center for Health Statistics Ethics Review Board approved the protocol. Written informed consent was obtained from all participants.

### Oxidative balance score

OBS is an index representing overall oxidative balance status based on 16 dietary nutrients (dietary fiber, carotene, riboflavin, niacin, vitamin B6, vitamin B12, vitamin C, vitamin E, total folate, calcium, magnesium, zinc, copper, selenium, total fat, iron) and 4 lifestyle indicators (physical activity, alcohol intake, cotinine and body mass index). The calculation of OBS was referred to previous studies (16, 17), which is illustrated in Supplementary Table S1. A total of 16 nutrients and alcohol intake were collected from two 24-h total dietary nutrient intakes files. NHANES participants were performed two 24-h dietary recall interviews. The first dietary recall was collected in-person during the NHANES visit, and the second recall was assessed through telephone follow-up 3 to 10 days later. Daily aggregates of food energy, nutrients, and food components from all foods were calculated using the U.S. Department of Agriculture (USDA)'s Food and Nutrient Database for Dietary Studies 2017–2018 and 2019–2020. We used the average of the two 24-h intakes of each nutrient to calculate the OBS (if only one 24-h record was available, this one-day value was used instead of the average). Physical activity was assessed derived from the questionnaire data using metabolic equivalent of task (MET) scores, which were calculated as NHANES suggested, including walking, bicycling, moderate and vigorous activity. Vigorous work-related and leisure-time activity were both assigned with a score coefficient of 8.0, while moderate work-related and leisure-time activity and walking or bicycling for transportation were assigned with a score coefficient of 4.0. Smoking was estimated by serum cotinine, which was measured by an isotope dilution-high performance liquid chromatography/atmospheric pressure chemical ionization tandem mass spectrometry (ID HPLC-APCI MS/MS) method. Body mass index (BMI) was collected from body measures and calculated as weight in kilograms divided by height in meters square. These components were divided into three groups by their sex-specific tertiles. Antioxidants (dietary fiber, carotene, riboflavin, niacin, vitamin B6, vitamin B12, vitamin C, vitamin E, total folate, calcium, magnesium, zinc, copper, selenium and physical activity) were assigned points from 0 to 2 for tertile groups 1 to 3 respectively, and pro-oxidants (total fat, iron, cotinine, alcohol intake, and BMI) were assigned points from 0 to 2 for tertile group 3 to 1 inversely. The total OBS was the sum of the antioxidant and pro-oxidant components, ranged from 0 to 40 points. Higher points indicate increased antioxidant levels.

### Outcome variables

Gallstones were determined by the medical history questionnaire. The positive response to the question “Has the doctor ever said you have gallstones?” was used to define gallstones. Conversely, the “no” response was defined as non-gallstones. Participants who refused to answer or do not know were excluded from our analysis.

### Covariates

Demographic data including age, gender, race/ethnicity, educational level, smoking status were collected. Race/ethnicity was categorized as Hispanic, non-Hispanic White, non-Hispanic Black, non-Hispanic Asian, or Other. Educational level was categorized as less than high school, high school, and more than high school. Comorbidity condition including hypertension, diabetes, coronary heart disease, malignant disease was recorded respectively, The definition of hypertension was required to meet one of these three criteria: (i) the mean systolic blood pressure ≥ 140 mmHg and / or diastolic blood pressure ≥ 90 mmHg measured 3 times on the same day if some blood pressure measurement data are missing, the value or average of the available measurement data was used; (ii) participants who take antihypertensive medication currently; (iii) self-reported hypertension in the questionnaire data. The definition of diabetes also needs to meet one of these criteria: (i) the glycated hemoglobin A1c (HbA1c) cut point of ≥6.5%; (ii) the FBG cut point of ≥126 mg/dL; (iii) participants who reported the diagnosis of diabetes or currently taking insulin or anti-diabetes drugs in the questionnaire date. The definition of coronary heart disease and malignant disease was both based on the medical history questionnaire. Serum parameters were extracted from laboratory data of NHANES, including total cholesterol, triglycerides, low-density lipoprotein, high-density lipoprotein, and fast blood glucose. Dietary inflammatory index (DII) was calculated by R package “dietaryindex” based on dietary intake data to reflecting overall dietary inflammatory potential as previously reported (18, 19). The triglyceride-glucose (TyG) index, a novel indicator of high atherosclerotic cardiovascular diseases risks, was calculated as Ln [fasting triglycerides (mg/dl) × fasting glucose (mg/dl)/^2^] (20).

According to previous literature, directed acyclic graph (DAG) was illustrated to show the relation among OBS, gallstones and potential confounders, which included age, gender, race/ethnicity, education, comorbidities, as shown in Supplementary Figure 1.

### Statistical analysis

All analyses were conducted in accordance with NHANES analysis and reporting criteria (21), and sample weights, clusters and stratification were all considered. The appropriate survey weight is based on the variable of interest that was collected on the smallest number of respondents (21). Multiple imputations were used for missing covariates including smoking status, comorbidities, and serum lipid levels. We applied the R “jomo” package to generate 10 imputed datasets after a burn-in of 500 iterations and 100 updates to ensure stochastic independence between imputed datasets, as described previously (22, 23).

Continuous variables are presented as mean (standard deviation) if they meet normal distribution or median (interquartile ranges) if not. Differences among different groups were compared using the weighted univariate linear regression and the Kruskal-Wallis test for normal continuous and nonnormal continuous variables, respectively. Categorical data were presented as frequencies (weighted percentages) and compared with chi-squared test.

Survey-weighted multiple logistic regression analysis was performed to investigate the association between OBS and gallstones. OBS was converted to a categorical variable by quartile and computed *p-*value for trend. Another three models for covariate adjustments were constructed to evaluate potential differences in the confounding effects. Model 2 was adjusted for age, gender, race/ethnicity and educational level. Model 3 was adjusted for the variables in Model 2 plus comorbidities. Model 4 was adjusted for the variables in Model 3 plus triglyceride.

To assess the effects of multiple components in OBS on gallstones, weighted quantile sum (WQS) regression model was used as reported previously (24–26). It can identify the contribution of each dietary or lifestyle components on the occurrence of gallstones. Participants were randomly divided into training (60%) and validation (40%) sets, and 1,000 bootstrap iterations on the training set were performed for WQS modeling. The R package “wqs” was used for WQS analysis.

Bayesian kernel machine regression (BKMR) model analysis was performed to examine the combined effect of individual component in OBS on predicted gallstone risk based on Gaussian process regression. As described previously (27), the posterior inclusion probability (PIP) was calculated to reflect the probability of overall OBS and specific component, respectively. Exposure variables with larger PIP values have more importance for the overall impact of gallstone risk. The BKMR model estimates were established after 20,000 iterations. The BKMR analysis was performed using R “bkmr” package.

We also used restricted cubic splines (RCS) models to explore the potential nonlinear associations between OBS and the prevalence of gallstones. Then, mediation effect analysis was performed to demonstrate the role of serum lipid levels in the relation between OBS and gallstones by using R package “mediation.”

In addition, sensitivity analysis was conducted to further examine the robustness of the findings. Firstly, as described above, using survey-weighted multiple logistic regression, WQS regression and BKMR model, the association between OBS and gallstones was assessed with and without the adjustment for possible confounding effects, and the single or joint effects of OBS components on the gallstones were also studied. Secondly, subgroup analyses and interaction tests were carried out to investigate whether different subgroups showed consistent trends with the overall population, and explore the effects and interactions of the covariates including age, gender, race/ethnicity, education and comorbidities. Thirdly, as OBS contains dietary and lifestyle components, we evaluated the impacts of dietary OBS and lifestyle OBS on gallstone disease respectively, in order to provide a more specific direction for gallstones prevention.

The *p* < 0.05 was considered statistically significant. All statistical analysis was performed by using R (version 4.2.2, R Foundation for Statistical Computing, Vienna, Austria).

## Results

### Study participants and baseline characteristics

In total, 15,560 participants were identified in NHANES 2017–2020. According to the inclusion criteria shown in Figure 1, 7,618 participants were finally included in this study, of whom 833 had gallstones. As listed in Table 1, there were significant differences between the gallstones group and non-gallstones group in terms of age, gender, race, education and smoking status. And individuals with gallstones had higher proportions with hypertension, diabetes, coronary heart disease and malignant disease. Serum cholesterol, triglyceride, high density lipoprotein-cholesterol and fasting blood glucose in gallstones group were more increased than those in non-gallstone group. Moreover, the metabolic risk index, triglyceride-glucose (TyG) values, were also significantly higher in gallstones group compared with non-gallstone group. From dietary data, individuals with gallstones had higher DII scores, which serves as a comprehensive index for dietary inflammation. Similarly, OBS in gallstone group was significantly lower than that in non-gallstone group (19.68 ± 6.61 vs. 20.48 ± 6.28, *p* = 0.029).

**Table 1:** Baseline characteristics of included participants.

Characteristics	Overall	Gallstones	No gallstones	*P*-value
Unweighted number	7,618	833	6,785	
Weighted number	237842359.48	25906309.27	211936050.22	
Age, mean (SD), years	48.62 (17.45)	58.07 (15.80)	47.46 (17.29)	<0.001
Gender, %				<0.001
Male	48.6	27.6	51.2	
Female	51.4	72.4	48.8	
Race/ethnicity, %				0.002
Hispanic	16.0	15.7	16.0	
Non-Hispanic White	62.4	66.8	61.9	
Non-Hispanic Black	11.6	7.9	12.0	
Non-Hispanic Asian	5.9	2.9	6.3	
Other	4.1	6.7	3.8	
Education, %				0.010
< High school	10.3	10.4	10.3	
High school	27.6	32.5	27.0	
> High school	62.0	57.1	62.7	
Smoking, %	42.6	47.1	42.0	0.026
Hypertension, %	42.7	62.0	40.3	<0.001
Diabetes, %	15.0	25.5	13.7	<0.001
Coronary heart disease, %	5.6	9.6	5.1	<0.001
Malignant disease, %	11.3	18.5	10.4	<0.001
BMI, mean (SD), kg/m^2^	29.75 (7.18)	33.21 (8.56)	29.33 (6.88)	<0.001
TC, mean (SD), mmol/L	4.87 (1.05)	4.85 (1.04)	4.87 (1.05)	0.782
TG, mean (SD), mmol/L	1.60 (1.13)	1.72 (1.01)	1.59 (1.14)	0.024
LDL-C, mean (SD), mmol/L	2.81 (0.94)	2.79 (0.96)	2.82 (0.93)	0.636
HDL-C, mean (SD), mmol/L	1.40 (0.46)	1.36 (0.44)	1.41 (0.46)	0.069
FBG, mean (SD), mg/dl	108.99 (34.76)	115.13 (35.68)	108.23 (34.57)	<0.001
TyG, mean (SD)	8.73 (0.70)	8.90 (0.64)	8.71 (0.71)	<0.001
DII, mean (SD)	1.15 (1.70)	1.42 (1.69)	1.12 (1.70)	0.001
Total OBS, mean (SD)	20.39 (6.32)	19.68 (6.61)	20.48 (6.28)	0.029

BMI, body mass index; TC, cholesterol; TG, triglyceride; LDL-C, low density lipoprotein-cholesterol; HDL-C, high density lipoprotein-cholesterol; FBG, fasting plasma glucose; TyG, triglyceride-glucose index, TyG = Ln (TG (mg/dl) * FBG (mg/dl) / 2); DII, dietary inflammatory index; OBS, oxidative balance score. Data were weighted estimate. Continuous data are presented as mean (SD). Categorical data are presented as weighted percentage (%).

### Association of OBS with gallstones

In logistics regression analysis, participants were grouped according to quantiles of OBS (Q1: 0–25%; Q2: 25–50%; Q3: 50–75%; Q4: 75–100%). Table 2 shows the association between total OBS and gallstones. As compared with the lowest quartile (Q1), the unadjusted model showed that higher OBS in Q2 and Q4 group were associated with lower gallstones risks. After adjusting for sociodemographic covariates and comorbidities in Model 2, Model 3 and Model 4, the Q2 and Q4 group of total OBS maintained a significant association with lower gallstones risks.

**Table 2:** Logistics regression analysis of the association between OBS and gallstones risks.

	Model 1		Model 2		Model 3		Model 4	
	OR (95% CI)	*P*	OR (95% CI)	*P*	OR (95% CI)	*P*	OR (95% CI)	*P*
OBS	0.98 (0.96–1.00)	0.030	0.99 (0.97–1.00)	0.12	0.99 (0.97–1.01)	0.2	0.99 (0.97–1.01)	0.2
Q1 (3.0–13.9)	–	–	–	–	–	–	–	–
Q2 (14.0–19.9)	0.71 (0.55–0.92)	0.012	0.72 (0.56–0.91)	0.009	0.73 (0.57–0.94)	0.019	0.72 (0.56–0.94)	0.018
Q3 (20.0–24.9)	0.78 (0.60–1.02)	0.065	0.79 (0.60–1.05)	0.10	0.83 (0.63–1.09)	0.2	0.82 (0.62–1.09)	0.2
Q4 (25.0–36.0)	0.65 (0.49–0.85)	0.003	0.69 (0.52–0.92)	0.015	0.72 (0.55–0.96)	0.026	0.72 (0.55–0.95)	0.023
Dietary OBS	0.99 (0.97–1.01)	0.200	0.99 (0.97–1.01)	0.4	0.99 (0.97–1.01)	0.4	0.99 (0.97–1.01)	0.4
Q1 (2.0–9.9)	–	–	–	–	–	–	–	–
Q2 (10.0–14.9)	0.77 (0.62–0.96)	0.023	0.72 (0.54–0.96)	0.030	0.73 (0.54–0.99)	0.045	0.73 (0.53–1.00)	0.052
Q3 (15.0–19.9)	0.78 (0.59–1.02)	0.066	0.78 (0.57–1.07)	0.11	0.80 (0.57–1.11)	0.15	0.80 (0.56–1.13)	0.200
Q4 (20.0–29.0)	0.87 (0.63–1.20)	0.400	0.93 (0.65–1.35)	0.700	0.96 (0.65–1.40)	0.800	0.95 (0.64–1.42)	0.800
Lifestyle OBS	0.87 (0.81–0.93)	<0.001	0.89 (0.80–0.99)	0.041	0.91 (0.81–1.02)	0.084	0.91 (0.81–1.03)	0.11
Q1 (0–2.9)	–	–	–	–	–	–	–	–
Q2 (3.0–3.9)	0.66 (0.49–0.88)	0.007	0.66 (0.47–0.92)	0.021	0.69 (0.48–0.99)	0.045	0.69 (0.47–1.02)	0.061
Q3 (4.0–5.9)	0.72 (0.51–1.00)	0.053	0.81 (0.53–1.25)	0.300	0.86 (0.52–1.41)	0.500	0.87 (0.52–1.46)	0.500
Q4 (6.0–8.0)	0.45 (0.26–0.79)	0.007	0.56 (0.28–1.13)	0.100	0.61 (0.29–1.31)	0.200	0.62 (0.28–1.41)	0.200

OR, odds ratio; CI, confidence interval; OBS, oxidative balance score. Model 1 had no covariate-adjusted. Model 2 adjusted age, gender, race, educational level. Model 3 adjusted age, gender, race, educational level, hypertension, diabetes, coronary heart disease, malignancy. Values are weighted OR (95% CI).

As OBS contains dietary and lifestyle components, we studied the association between dietary OBS, lifestyle OBS and gallstone risks, respectively, (Table 2). The unadjusted model showed that dietary OBS in Q2 and Q3 group and lifestyle OBS in Q2, Q3 and Q4 group were associated with lower gallstones risks. And after adjusting for sociodemographic covariates and comorbidities, dietary and lifestyle OBS both in Q2 group remained significantly associated with lower risk of gallstones.

**Figure 2:**
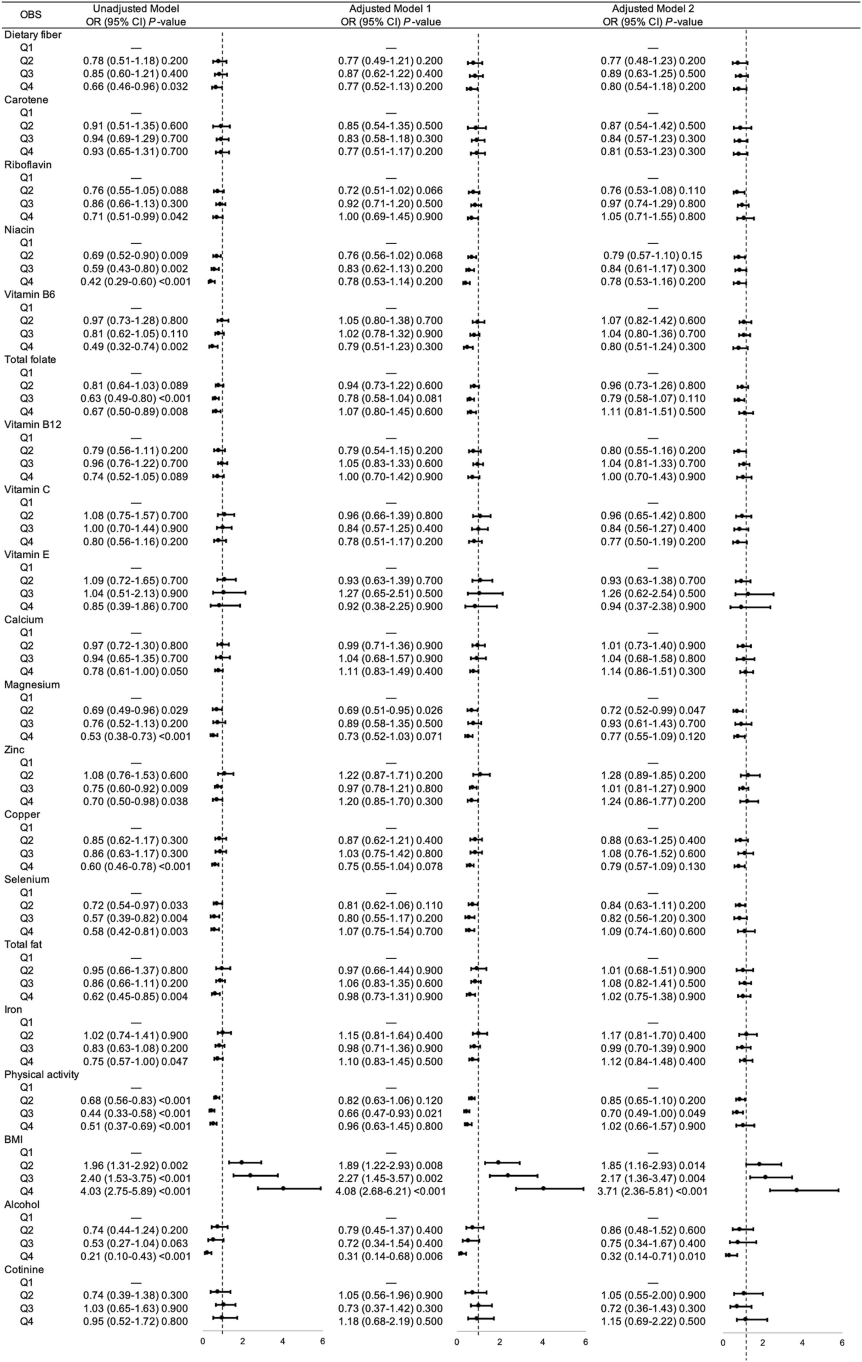
Unadjusted and adjusted associations between 20 OBS components and gallstones. Adjusted model = multivariable logistic regression model. Adjusted model 1 was multivariable logistic regression model adjusted age, gender, race, educational level. Adjusted model 2 was multivariable logistic regression model adjusted age, gender, race, educational level, hypertension, diabetes, coronary heart disease, malignancy. OR, odds ratio; CI, confidence interval; OBS, oxidative balance score; BMI, body mass index.

Then we analyzed the association between each components in OBS and gallstones risks, respectively. As shown in Figure 2, there were significant correlation between most components and gallstone risks, while after adjusting for sociodemographic covariates, niacin (Q2), total folate (Q3), magnesium (Q2), physical activity (Q3), BMI (Q2, Q3, Q4), alcohol intake (Q4) remained associated with decreased risk of gallstones.

**Figure 3:**
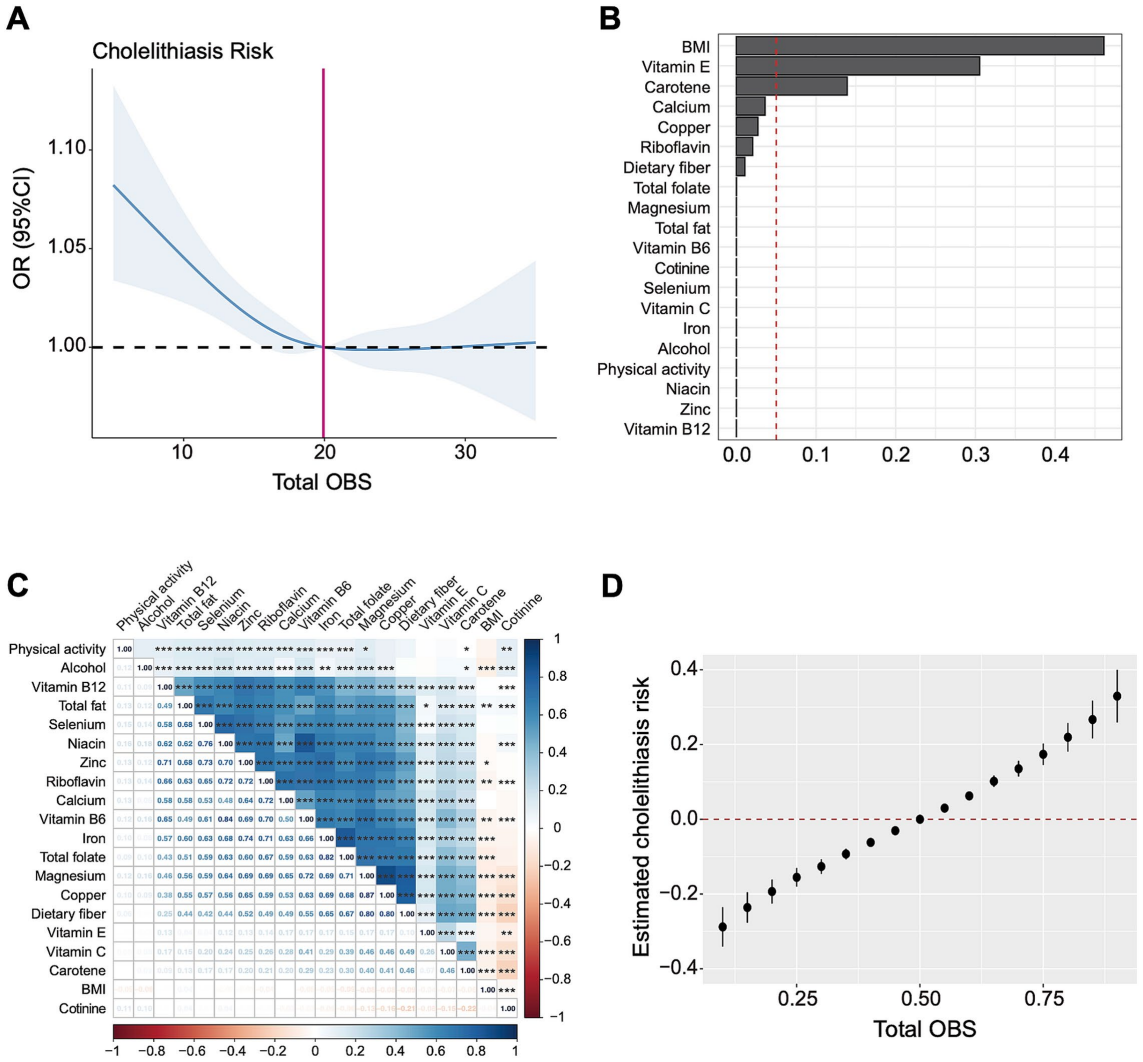
The association between OBS and gallstones based on RCS, WQS, and BKMR analysis. **(A)** Restricted cubic spline analysis revealed the non-linear relationship between OBS and gallstones. **(B)** The WQS model showed estimated weighted values of 20 OBS components for gallstones risk. **(C)** Matrix heatmap of correlations among 20 components in OBS. **(D)** The BKMR model showed the joint effect (95% CI) of OBS components on gallstones when all components were at particular percentiles compared with all components at their 50th percentile. The BKMR model was adjusted for age, gender, race/ethnicity and educational level. OR, odds ratio; CI, confidence interval; OBS, oxidative balance score; BMI, body mass index.

Furthermore, restricted cubic spline curves (Figure 3A) visualized the non-linear relationship between the OBS and gallstones (overall *p* = 0.0003, nonlinear *p* = 0.03). Under the premise of the total OBS below 19.92, a lower OBS was associated with increased risk of gallstone disease, whereas gallstone risk remained stable when the OBS exceeded 19.92.

### Sub-analysis in the association of OBS with gallstones

**Figure 4:**
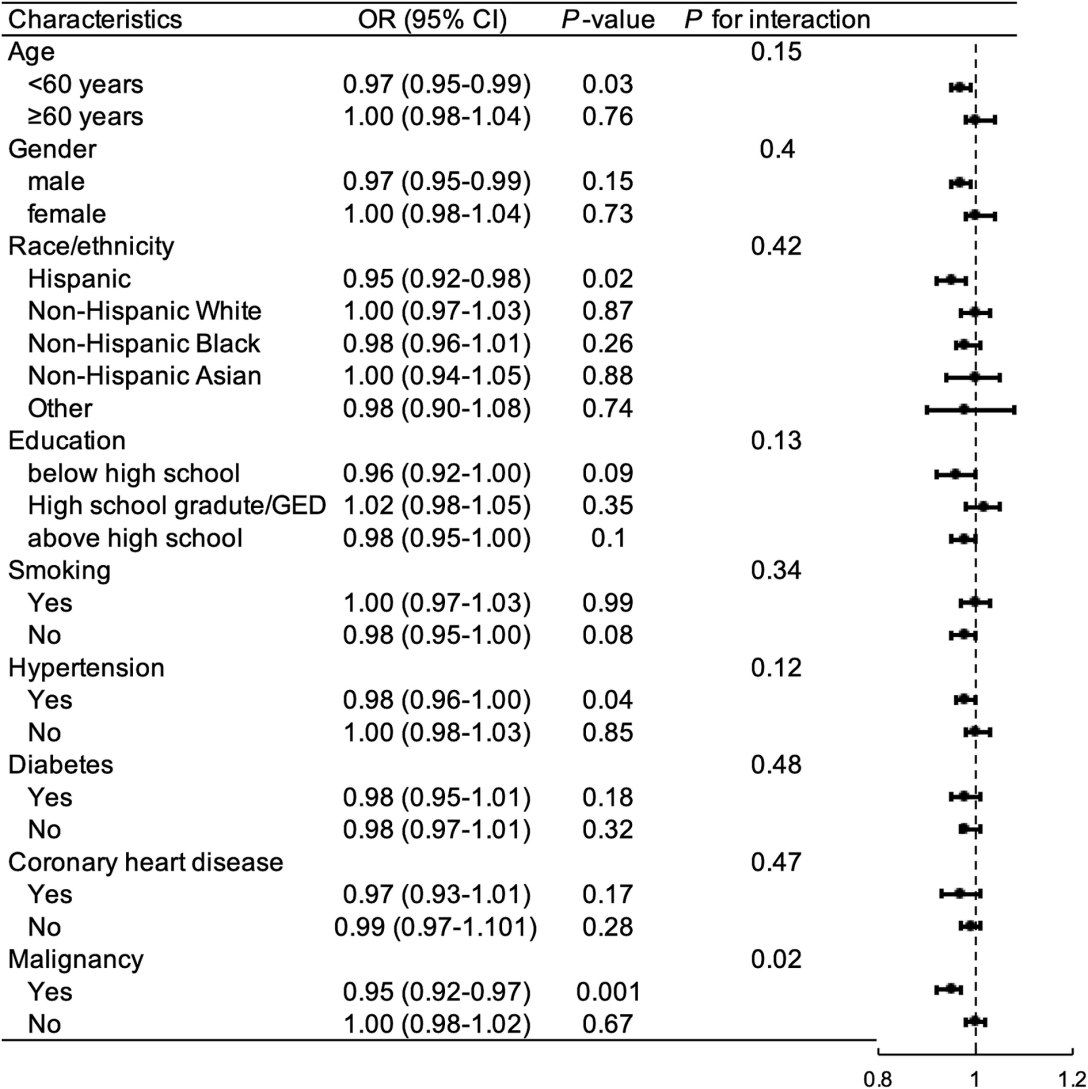
Subgroup and interaction analysis to assess the association between OBS and gallstones. OR, odds ratio; CI, confidence interval; GED, general educational development; OBS, oxidative balance score.

Subgroup analyses were performed to examine whether the relationship between OBS and gallstones was influenced by covariables. As illustrated in Figure 4, after controlling for selected confounding factors, increased total OBS exhibited a significant association with a higher risk of gallstones in participants who were under 60 years old, Hispanic, educated below high school, non-smokers, had hypertension or malignancy.

The interaction between malignancy and the relationship between OBS and gallstone was significant (*P* for interaction = 0.008). Among the other subgroups, there were no significant interactions between OBS and gallstone incidence (*P* for interaction >0.05).

### WQS and BKMR analysis on the relation of overall OBS components and gallstones

WQS regression analysis indicated a negative association between 20 OBS components and gallstones risk (OR = 2.21, 95% CI 1.84–2.66, *p* < 0.001). Figure 3B showed the estimated weights of each OBS components for the WQS model, in which the top three highest weighted components in the WQS model were BMI (46.1%), vitamin E (30.5%), and carotene (13.9%).

Through correlation analysis, there were several significant correlations among 20 components in OBS, as shown in Figure 3C. We further applied the BKMR model to analyze the joint effect of OBS components on the risk of gallstones, which indicated a positive association between total OBS and gallstones (Figure 3D). Compared to all OBS components at the 50^th^ percentile, the risk of gallstones significantly increased when OBS components were at the 55th percentile or above, and significantly decreased when all OBS components were at the 45th percentile or lower. The top three highest PIP values generated from the BKMR model were BMI (1.0), magnesium (0.0246), vitamin B6 (0.0178). Consistently, the estimated univariate exposure-response function of each component was demonstrated in Supplementary Figure 2, BMI was positively correlated with gallstone risk when the other components were fixed at the median.

### Mediation analysis of serum lipid on the association between OBS and gallstones

**Figure 5:**
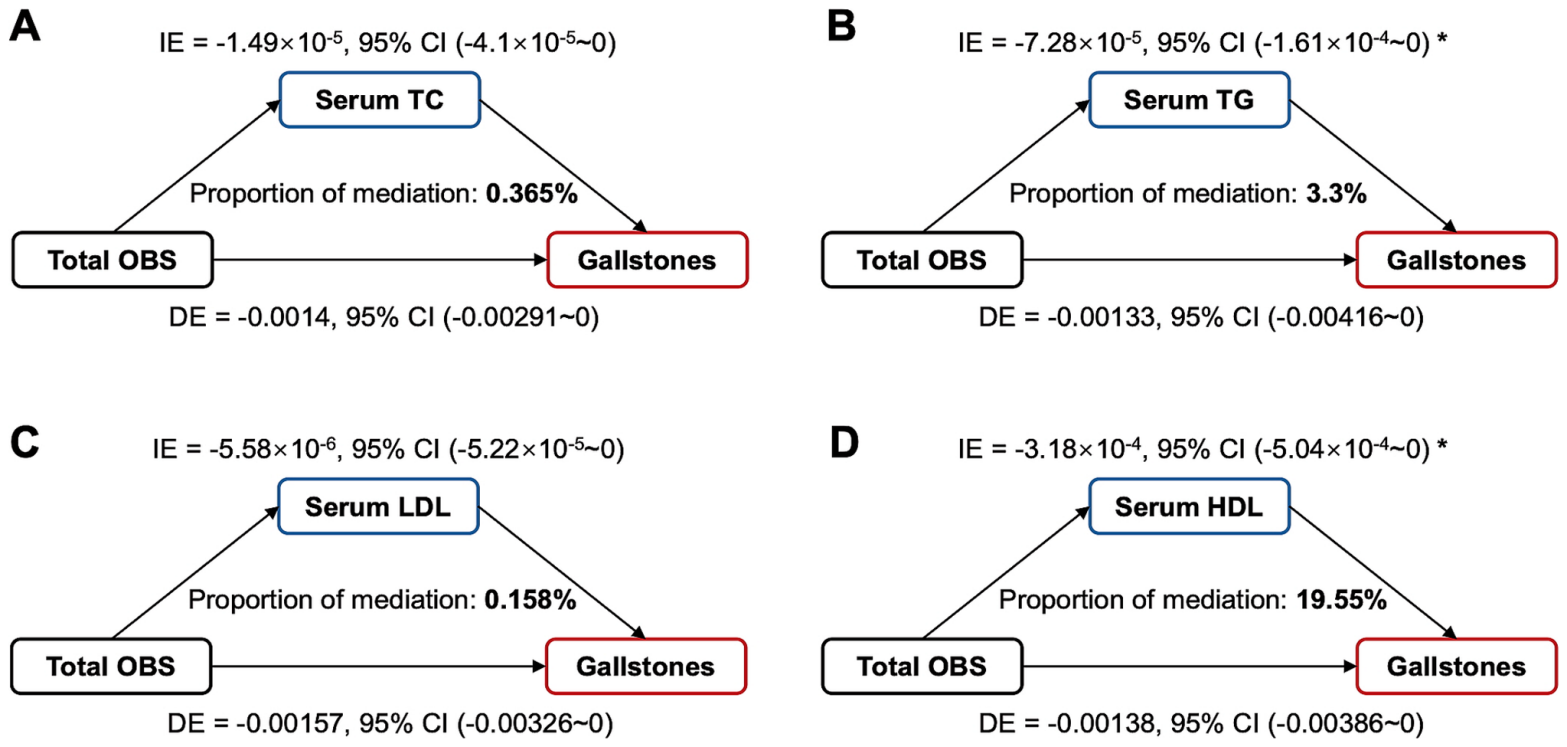
Estimated proportion of the association between total OBS and gallstones mediated by serum lipid levels. **(A)** TC, cholesterol; **(B)** TG, triglyceride; **(C)** LDL, low-density lipoprotein cholesterol; **(D)** HDL, high-density lipoprotein cholesterol; Mediation analysis adjusted age, gender, race, educational level, hypertension, diabetes, coronary heart disease. OBS, oxidative balance score; IE, indirect effect; DE, direct effect; Proportion of mediation = IE / (DE + IE); * *p* < 0.05.

We further evaluated whether serum lipid levels mediate the association between OBS and gallstones. As shown in Figure 5, the mediating effect of TG and HDL on the association was 3.3% and 19.55%, respectively, and there were no significant mediating effects of serum TC and LDL. And moreover, TG (OR = 1.09, 95% CI 1.01–1.18, *p* = 0.022) and HDL (OR = 0.80, 95% CI 0.62–1.03, *p* = 0.077) were correlated with gallstone risks. Serum TG levels were negatively associated with OBS, which were, respectively, 1.60 (1.08), 1.70 (1.23), 1.58 (1.13), 1.53 (1.06) in Q1 to Q4 group of total OBS (*p* = 0.002). Serum HDL levels were positively associated with OBS, which were, respectively, 1.36 (0.48), 1.38 (0.46), 1.41 (0.46), 1.44 (0.44) in Q1 to Q4 group of total OBS (*p* = 0.008).

## Discussion

This cross-sectional study revealed a significant and non-linear relationship between OBS and gallstones risk in US general population. Higher dietary and lifestyle OBS, indicating increased antioxidant levels, was associated with lower gallstones risk. BMI, vitamin E, vitamin B6, magnesium and carotene were identified as having relatively important role in WQS and BKMR model. Serum TG and HDL levels were further identified as partially mediating the association between OBS and gallstones.

OBS represents an individual’s oxidative stress status and has been reported to have associated with metabolic diseases (11, 28, 29) or inflammatory condition (12, 30). Until now, there has no study about OBS and gallstone disease. From the perspective of oxidative stress, there were some evidence about the role of oxidative stress in pathogenesis of gallstones. Metagenomic sequencing of bile from gallstone patients showed significantly enriched pathways related to glycerophospholipid and glutathione metabolism, which are involved in inflammation and oxidative stress responses (31). The oxidative stress marker 8-OH-dG, was found to significantly increased at plasma and DNA level in patients with cholelithiasis (32). Kuba et al. (33) reported that a microsomal enzyme, Elovl6, can regulate fatty acid metabolism and hepatic oxidative stress, leading to increased formation of cholesterol crystals. Additionally, it was proved that oxidized bilirubin free radical existed in the gallstones using electron paramagnetic resonance technique, which promoted stone nucleation and deposition (7). These observations suggested that oxidative stress is closely related to the development of gallstones, which were consistent with our findings that the index of anti-oxidant capacity, OBS, was strongly associated with gallstones.

Specifically, OBS was derived from multiple dietary and lifestyle components. Our results showed dietary OBS and lifestyle OBS were both associated with gallstones risk. A Iranian case–control study demonstrated that higher dietary total antioxidant capacity had lower risk for gallstone disease (34), which is consistent with our results. Previous studies have proved several dietary nutrients exert anti-oxidative functions and protective effect on gallstone disease. Vitamin E, as an antioxidant, might suppress the gallstone formation. A cross-sectional study from Germany revealed that low proportions of gallstone disease were observed in the top tertile of plasma *α*-tocopherol/cholesterol ratio group (35). The reduction of oxidative stress after vitamin E supplementation may improve lipid metabolism via gene expression regulation, involving in the cholesterol synthesis pathway, lipid transport, lipogenesis, and mitochondrial biogenesis (36, 37). B vitamins, especially vitamin B6, have been identified in relation to hypercholesterolemia or calculus of kidney (38), which partially supports our findings. Previous studies and our results both found magnesium intake had negative association with gallstones risk (39, 40). Magnesium may alter biliary cholesterol saturation and enhance gallbladder emptying, and lack of magnesium has been found to increase levels of triglycerides and decreasing levels of HDL-C in the bloodstream simultaneously, which can explain our serum lipid changes (39, 41). In addition, different dietary habits have been proved to affect biliary microbial composition and gallstone development (42). On the other hand, in our study, obesity is the most significant risk factor associated with gallstones. Obesity is a current health problem globally and is a recognized risk factor for the development of metabolic comorbidities, and normal BMI can promote the maintenance of a proper nutritional status (43). The abnormalities of adipocyte function and adipocytokines contributed to the local and systemic inflammation responses and increased oxidative stress in the context of obesity (44). Mechanistically, lipid metabolism can be regulated by inflammatory pathways, oxidative stress or gut dysbiosis, which are always accompanied by obesity (45, 46). Multiple proteins in bile samples, regulating the inflammatory and metabolic processes, were proved to have differential expression between the obese and non-obese individuals (47). Increased cholesterol supersaturation and nucleating factors in bile promote the formation of gallstones, especially cholesterol stones. Moreover, obesity also causes abnormalities in gastrointestinal hormones secretion. For example, ghrelin, mainly produced by the gastric fundus, has been generally regarded as a key regulator on obesity (48). Previous studies revealed that ghrelin might had a potential role on glucose regulation and was associated with gallstone disease (48, 49).

The subgroup analysis showed differences in the association between OBS and gallstones across population characteristics. Individuals aged <60 years appeared to be more sensitive to OBS on gallstone than older participants (≥60 years). Young population tend to have unhealthy dietary and lifestyle habits compare to the elderly, so OBS increase may be particularly beneficial to these young participants. And the elderly are more likely to have metabolic disorders, making it difficult to increase antioxidant capacity simply through improving exogenous diet and lifestyle. Our results also indicated that Hispanic and individuals with lower education level were more susceptible to the effects of OBS on gallstones. Epidemiological studies have shown gallstones affected up to 30% of Hispanic populations in Central and South America, for which genetic factors can account predominantly (50). In addition, gallstone risks in participants with metabolic or malignant comorbidities are more prone to be influenced by OBS. Previous studies showed that gallstone disease was significantly related with cardiometabolic risks (28), metabolic syndrome (OR 1.31, *p* = 0.02) (51) and malignancy (OR 1.3, *p* < 0.001) (52). Nutrients metabolism can affected cholesterol metabolism and cardiometabolic disease risks by oxidative stress related pathway (53). These might partially explain why gallstones risk in patients with metabolic diseases is relatively significantly affected by OBS compared with those without metabolic comorbidities.

Furthermore, serum TG and HDL partially mediated the association between OBS and gallstones in our study, which suggested that serum TG and HDL levels might be influenced by oxidative stress and can be regarded as key indicators in clinical monitoring of gallstones. As for HDL, the interaction of HDL and oxidative stress is always bi-directional and multifactorial. HDL has been demonstrated to possess anti-oxidant activity and can mediate the inhibition of LDL oxidation, and in return, increased oxidative stress may affect the structural state of HDL-C (54–57). Furlong et al. (58) reported that HDL-related enzyme paraoxonase-3 knockout mice exhibited susceptible to gallstone formation and metabolic dysfunction through regulation of oxidative stress and inflammatory response. Thus HDL may serve as a reflection of oxidative capacity or partially exert its antioxidant activity. And for TG, recent study revealed that chronic oxidative stress induces TG accumulation by regulating the expression of lipopolysaccharide-binding protein and lipid-redox homeostasis (59). Zhang et al. (60) reported that peroxisome proliferator-activated receptor (PPAR) *α* signaling pathway, involved in oxidative stress and inflammatory response, plays an important role in the synthesis of TG. The relationship between oxidative stress and lipid metabolism may partly explains the association between OBS and gallstones.

From this study, we hope the OBS can provide great practical value for gallstone assessment in clinical work. First, considering its convenience and cost-effectiveness, the OBS can be used as a preliminary evaluation for gallstone risk in the healthy check-ups. From our results, it is recommended that consequent examinations (e.g., the abdominal ultrasound) should be arranged to screen for gallstones for the individuals with the OBS below 19.92 or lower. Besides, as for the patients with gallstones or high gallstones risks (e.g., low OBS, hyperlipidemia, etc.), the measurement of OBS can provide a more accurate strategy for improving or preventing the gallstones, including specific daily food adjustments, quantified exercise intensity and weight management regimen.

To our knowledge, this is the first study to comprehensively analyze the association between OBS and gallstones through multiple statistical models. We minimize confounding bias via adjustment for multiple covariates and subgroup and interaction analysis. Then we investigated the relationship between each component of OBS and gallstones risk to reflect the most critical factors in OBS. Moreover, mediation analysis in this study found that serum lipid may plays a mediating role between OBS and gallstones, which indicates the potential relation among lipid metabolism, oxidative stress and cholelithiasis and also provides theoretical evidence for clinical surveillance.

This study also has some limitations. Firstly, gallstone compositions were not available in the NHANES data, so the role of OBS in gallstones with different compositions is not clear. And there are also other potential confounding factors that have not been assessed in this study, for instance, patients with genetic predisposition, bariatric surgery-induced rapid weight loss, or hematological disorders, may predispose to gallstones. Secondly, despite thorough sensitivity analysis in this study, the selection bias still remains because of the exclusion of participants with missing data. Thirdly, recall bias was inevitable as comorbidities and OBS data were acquired from questionnaire. And the cross-sectional characteristics of this study cannot evaluate the causal relations between OBS and gallstones. Thus more prospective cohort studies are required to validate the role of OBS in gallstone disease.

In conclusion, increased oxidative balance, as evidenced by a higher OBS, are positively associated with reduction of gallstone risk. These findings highlight the previously unknown associations between dietary and lifestyle oxidative levels and gallstones in the general population. Surveillance for serum lipid or nutrients and early interventions targeting for antioxidant-rich diets or lifestyles may be helpful for gallstone prevention. Further prospective and experimental studies are warranted to confirm causal relationship and potential mechanisms.

## Data Availability

The datasets presented in this study can be found in online repositories. The names of the repository/repositories and accession number(s) can be found at: https://www.cdc.gov/nchs/nhanes/.
